# Correlation Between the Age at Menarche, Time to Diagnosis, and Disease Severity Assessed Using the rASRM and #ENZIAN Classifications in Patients Diagnosed with Advanced Endometriosis

**DOI:** 10.3390/jcm15197526

**Published:** 2026-09-27

**Authors:** Krzysztof Przyśliwski, Bogumił Paweł Siekierski, Maciej Pliszkiewicz, Joanna Jacko, Anna Bogaczyk, Wojciech Kluz, Tomasz Kluz

**Affiliations:** 1 Faculty of Health Sciences and Physiology, University Center for Research and Development in Health Sciences, Collegium Medicum, Rzeszów University, Ul. Warzywna 1a, Bud. G5, G4, 35-310 Rzeszow, Poland; 2 Medicover Wilanów Hospital, Medicover Healthcare Services, Al. Rzeczypospolitej 5, 02-972 Warsaw, Poland; 3 Department of Gynecology, Gynecology Oncology and Obstetrics, Faculty of Medicine, University of Rzeszów, Al. Tadeusza Rejtana 16c, 35-959 Rzeszow, Poland; 4 Faculty of Medicine and Health Sciences, Andrzej Frycz Modrzewski Krakow University, Gustawa Herlinga-Grudzińskiego 1, 30-705 Krakow, Poland

**Keywords:** endometriosis, menarche, rASRM classification, #ENZIAN classification, diagnostic delay in endometriosis, disease severity

## Abstract

**Background/Objectives**: Endometriosis is a chronic inflammatory gynecological condition characterized by a highly variable clinical picture, with pain at multiple sites being among its most disabling features. Women are mostly diagnosed between 20 and 45 years of age, but the time from symptom onset to diagnosis varies across countries, from a few months to more than a decade. Symptoms unequivocally linked to disease onset are difficult to identify, since hormonal activity during puberty varies between individuals. This study assessed the relationship between the age at menarche and the duration, regularity, heaviness, and painfulness of the first menstrual bleedings and the occurrence and severity of endometriosis. **Methods**: This retrospective study included 188 patients with advanced endometriosis qualified for surgical excision of endometriotic lesions between 1 May 2024 and 31 May 2026. Data were obtained through a detailed history taken as part of a preoperative questionnaire. The severity of endometriosis was assessed intraoperatively according to rASRM and #ENZIAN systems. Validated statistical tests were applied, with *p*-value < 0.05 considered significant. **Results**: No significant correlation was found between the age at menarche and the duration, regularity, or heaviness of the first menstrual bleeding and rASRM-assessed severity. A positive correlation was observed between symptom duration, in years, and first-menstruation pain intensity, and between later menarche and #ENZIAN P and T localization. **Conclusions**: The age at menarche, disease duration, and diagnostic delay do not consistently correlate with rASRM-assessed severity. These findings should be interpreted with caution given the selected cohort and may not generalize to patients with milder disease or those managed non-surgically.

## 1. Introduction

It is estimated that endometriosis affects between 10% and 15% of women of reproductive age [1,2], with prevalence rising to as much as 70% among women presenting with chronic pelvic pain [3]. Globally, the disease is thought to affect between 176 and 190 million women [4,5], and roughly one in three of these women experiences infertility [6].

Diagnosis typically occurs between 20 and 45 years of age, with reported median and mean ages of 28.8 and 29.4 ± 7.7 years, respectively [7]. The condition is defined by ectopic endometrial-like tissue implanted outside the uterine cavity, predominantly within the peritoneal cavity, where cyclical hormonal stimulation drives a sustained inflammatory and proliferative process [8,9].

The main symptoms of endometriosis include painful menstruation (dysmenorrhea), lower abdominal pain, pain during urination (dysuria), pain during defecation (dyschezia), pain during intercourse (dyspareunia), infertility [10,11], abnormal uterine bleeding, and fatigue [12]. While roughly one-fifth to one-quarter of affected women remain asymptomatic, up to 80% of symptomatic patients report chronic pain, and fertility problems are documented in 30–50% of cases [13,14].

Substantial diagnostic delay remains characteristic of endometriosis, driven by the heterogeneity and variable severity of symptoms combined with underused or unavailable diagnostic tools. Reported intervals between symptom onset and confirmed diagnosis span from a few years to well over a decade, varying with population characteristics and the diagnostic criteria applied. Following De Corte et al. [15], this interval can be conceptually split into a primary diagnosis time—from symptom onset to first medical contact—and a clinical diagnosis time—from that first contact to definitive diagnosis.

The aim of this retrospective study was to investigate correlations between the age at menarche, categorized into age groups (7–11 years; 12–14 years; 15–17 years), the characteristics of the first menstrual bleedings in terms of regularity, pain intensity, and heaviness, and the patient’s age at diagnosis, as well as the number of years of symptoms associated with endometriosis (overall diagnosis time) and disease severity. We hypothesized that an earlier age at menarche would be associated with more severe endometriosis, as assessed by the rASRM and #ENZIAN classifications, and with a longer diagnostic delay.

## 2. Materials and Methods

### 2.1. Study Population

This retrospective study enrolled 188 women scheduled for laparoscopic excision of advanced deep infiltrating endometriosis lesions at the Department of Gynecology, Medicover Hospital, Warsaw, between 1 May 2024 and 31 May 2026—the same surgical cohort and center previously described by our group in the context of pain characteristics and intraoperative staging [16]. Diagnosis was established by transvaginal ultrasonography (TVUS), supplemented where necessary by pelvic magnetic resonance imaging (MRI) and clinical evaluation.

The study population represented a selected cohort of patients with advanced (deep) endometriosis (DE) referred for surgical treatment at a specialized care center. The sample size was not determined based on a priori power calculations; instead, it reflects a sample representative of real-world clinical practice. Given the exploratory nature of this study and its relatively limited sample size, no formal a priori or post-hoc statistical power analysis was performed, and the minimum detectable effect sizes were therefore not calculated. The results should therefore be interpreted with caution and regarded as hypothesis-generating rather than confirmatory.

Before enrollment, all participants received written information on the aim of this study and provided informed consent to participate. The questionnaire survey was part of a broader, ongoing study evaluating the quality of life of patients with endometriosis before and after surgery, which was approved by the Bioethics Committee of the Regional Medical Chamber in Warsaw (approval no. KB/1472/23).

Inclusion criteria comprised a diagnosis of deep infiltrating endometriosis in patients qualifying for surgical treatment, age over 18 years, and written informed consent to participate in the study. Exclusion criteria included age under 18 years, refusal to participate, or disqualification from surgical treatment for any reason.

Consistent with our previous work using this cohort [16], disease severity was staged intraoperatively using two complementary systems: the four-stage revised American Society for Reproductive Medicine classification (rASRM) and the #ENZIAN system for deep infiltrating endometriosis developed by the Stiftung Endometriose-Forschung (SEF).

The surgical team assessing intraoperative disease severity according to the rASRM and #ENZIAN classifications was blinded to the questionnaire data, including the patients’ menstrual history. Inter-rater reliability for the #ENZIAN classifications was not formally assessed.

### 2.2. Data Collection

Sociodemographic data and detailed medical history were collected using an original questionnaire developed specifically for this study. Demographic variables included age, level of education, employment status, and predominant body position during work. Clinical variables included age, height, body weight, characteristics of the first menstrual bleeding (date of onset, heaviness, regularity, and associated pain), as well as the regularity, intensity, and pain associated with subsequent menstrual cycles.

The questionnaire also included questions regarding the age, self-reported by the patient, at which the first symptoms she associates with disease onset appeared, the number of years from disease onset to diagnosis, and the age at which the diagnosis was made.

Additional data on the frequency, duration, and intensity of pain at various locations, questions assessing the impact of pain severity on daily functioning, the need for analgesics, the need for medical interventions due to pain, the types of therapies used to date, and the number of endometriosis-related surgical procedures were omitted from this analysis, as pain-related characteristics of these patients have already been reported in our previous publication [16], which focused on the relationship between pain intensity and intraoperative disease stage. The present study instead focuses on menarche characteristics, diagnostic delay, and disease severity.

The diagnostic difficulties arising from the multiplicity of symptoms and the disease course result in a substantial prolongation of the time between the onset of the first symptoms that can be linked to endometriosis and the time of diagnosis. The interval varies depending on the country, population characteristics, inclusion criteria, and the definition adopted.

The study focused on identifying correlations between age at menarche and its characteristics within age groups (7–11 years; 12–14 years; 15–17 years), and the patient’s age at diagnosis, as well as the number of years of symptoms associated with endometriosis (overall diagnosis time) and disease severity.

Disease staging was carried out during surgery by the gynecological team at Medicover Hospital, applying the rASRM and #ENZIAN grading systems [16,17,18]. The severity data analyzed in this study were subsequently retrieved from the corresponding operative reports.

### 2.3. Statistical Analysis

Data obtained from the original questionnaire, including the sociodemographic section and detailed history data, were entered into a specially prepared Microsoft Excel spreadsheet and subsequently subjected to statistical analysis.

Descriptive statistics for numerical variables comprised means, standard deviations, medians, quartiles, and observed ranges, while categorical variables were summarized as absolute and relative (percentage) frequencies.

Between-group comparisons followed a consistent scheme, applied identically to that used in our earlier analysis of this cohort [16]: categorical variables were compared via the chi-squared test (Yates-corrected for 2 × 2 tables), switching to Fisher’s exact test whenever expected cell counts were insufficient; two-group comparisons of numerical variables relied on the Mann–Whitney U test, and comparisons across three or more groups used the Kruskal–Wallis test, followed by Dunn’s post-hoc test in cases where statistically significant differences between groups were detected.

A significance level of 0.05 was adopted for the analysis; thus, all *p*-values below 0.05 were interpreted as indicating significant associations.

The analysis was performed using R, version 4.6.0 [19].

To assess the overall risk of false-positive findings arising from the large number of subgroup comparisons, the Benjamini–Hochberg procedure was applied to all reported *p*-values, and false discovery rate (FDR)—adjusted q-values were calculated.

## 3. Results

The following analyses should be regarded as preliminary, given the number of subgroup comparisons performed. The analysis addressed:

Correlations between the patient’s age at menarche and the patient’s age at which the first symptoms she associates with endometriosis appeared.

Correlations between the patient’s age at menarche and disease severity as assessed by the rASRM classification.

Correlations between the patient’s age at diagnosis and disease severity according to the rASRM scale and lesion localization according to the #ENZIAN scale.

Correlations between the duration of symptoms currently attributed to endometriosis and the time from first presenting to a physician with initial symptoms to the diagnosis of endometriosis and disease severity according to the rASRM scale and lesion localization according to the #ENZIAN scale.

Correlations between the regularity, heaviness, and painfulness of the first menstrual bleedings and the occurrence of the first symptoms that the patient associates with the onset of endometriosis.

### 3.1. Baseline Characteristics

The final analyzed cohort comprised 188 women with a diagnosis of advanced endometriosis who underwent elective laparoscopic excision at Medicover Hospital, Warsaw. Endometriosis was histologically confirmed in every case, and all participants fulfilled the inclusion criteria and had signed the informed consent form.

### 3.2. Detailed Medical Interview

Detailed interview data were available for the full study cohort (N = 188), with missing values appropriately handled within the baseline dataset prior to statistical analysis. Table 1 summarizes the demographic and clinical characteristics of the patient group. As every subsequent comparison draws on this same set of patients, these baseline figures are presented only once and are not reproduced in the tables that follow.

### 3.3. Correlation Between the Patient’s Age at Menarche and the Patient’s Age at Onset of the First Symptoms Associated with Endometriosis

Correlation between the age at menarche, categorized into age groups (7–11 years, 12–14 years, and 15–17 years), and the age at diagnosis, considering the overall diagnosis time and the primary diagnosis time.

No significant differences were found between the groups (*p*-value > 0.05) (Table 2, Figure 1).

### 3.4. Correlation Between the Patient’s Age at Menarche and Disease Severity as Assessed by the rASRM and #ENZIAN Classifications

The general rule adopted for the analysis was that the groups of fewer than 5 individuals were merged with the other groups (Table 3, Figure 2). Groups defined by the age at diagnosis of endometriosis were selected so that each contained a similar number of patients.

*p*-values below 0.05 indicated statistically significant differences between groups.

**Menarche vs. #ENZIAN P:**

The “P” score was highest among patients who reached menarche at 15–17 years of age, and lowest among patients who reached menarche at 7–11 years of age.

**Menarche vs. #ENZIAN T:**

The “T” score was highest among patients who reached menarche at 15–17 years of age, and lowest among patients who reached menarche at 7–11 years of age.

### 3.5. Correlation Between the Patient’s Age at Diagnosis and Disease Severity According to the rASRM and #ENZIAN Classifications

No significant differences were found between the groups (all *p*-value > 0.05) (Table 4, Figure 3).

### 3.6. Duration of Symptoms Currently Attributed to Endometriosis and Disease Severity

No significant differences were found between the groups (all *p*-value > 0.05) (Table 5, Figure 4).

### 3.7. The Time from First Presenting to a Physician with Initial Symptoms to the Diagnosis of Endometriosis

No significant differences were found between the groups (all *p*-value > 0.05) (Table 6, Figure 5).

### 3.8. Regularity, Heaviness, and Painfulness of the First Menstrual Bleedings, and the Onset of the First Symptoms the Patient Associates with Endometriosis

*p*-values below 0.05 indicate statistically significant differences between groups.

**Painfulness of the First Menstrual Bleedings:**

The duration of symptoms currently attributed to endometriosis was longest among patients whose first menstrual bleedings were associated with pain that was difficult to tolerate, and shortest among patients whose first menstrual bleedings were painless (Table 7, Figure 6).

## 4. Discussion

The available databases contain numerous publications on the causes of endometriosis and the assessment of the risk of its occurrence, depending on various measurable (objective) and non-measurable (subjective) parameters related to genetic predisposition [20], development factors [21], and modifying factors such as lifestyle [22], cultural determinants [23], sociodemographic factors [24], and economic factors [25].

One of the important aspects related to the course of the disease and its impact on patients’ physical and mental health [26] is the timing of symptom onset and their severity. The chronic nature of the disease, the cyclical pattern of symptoms, symptom intensity, and the effectiveness of pharmacological and non-pharmacological therapies also influence patients’ determination to seek a solution to their health problems. This determination, together with health awareness and the knowledge of medical professionals, ultimately determines the time to correct diagnosis.

Endometriosis is diagnosed in women across a wide age range, most commonly between 20 and 40 years of age, but it may already be diagnosed in adolescents as well as in postmenopausal women [27,28]. The time from symptom onset to diagnosis varies considerably and, according to reports, ranges from approximately 0.3 to 12 years [29]. In our study, the mean age at which the diagnosis was made by a physician was 32.47 years. The study methodology defined the primary diagnosis time and the overall diagnosis time using the following intervals: <12 months, 1–3 years, 4–6 years, 7–9 years, and ≥10 years. Among the patients included in the study, these times were, respectively, 10 years or more in 57.45%, 7–9 years in 12.3%, 4–6 years in a further 15.96%, 1–3 years in 12.3%, and less than 12 months in only 2.13%. Given the adopted methodology, the results obtained should be interpreted with caution and regarded as approximate rather than definitive. For this reason, the results cannot be directly compared with data published by other authors between 2018 and 2023. It should be noted that the mean time in Poland is longer than data published in 2020 for Australia (2020—diagnostic delay: 8.1 years [30]), the UK (2020—8 years), New Zealand (2022—8.7 years [31]), and Germany (2020—8.6 years [32]); markedly longer than in Canada (2020—5.4 years [33]); and closely comparable to results from France (2022—12 years [34]) and Italy (11.4 years [35]). The most frequently reported causes of diagnostic delay in the literature include patient-related factors, physician-related factors, and delays related to the healthcare system and payer [36].

The clinical consequences of such prolonged diagnostic delay are substantial. A longer interval between symptom onset and diagnosis has been associated with disease progression, including the development of more extensive and multi-organ involvement, which may in turn reduce the likelihood of achieving complete surgical resection and increase perioperative risk. Beyond disease progression, prolonged diagnostic uncertainty is associated with a considerable psychosocial burden, including chronic pain, anxiety, depression, and impaired quality of life, as repeatedly reported in the endometriosis literature [25,26,29].

From a health-system perspective, delayed diagnosis is also associated with increased healthcare utilization, including repeated consultations, diagnostic procedures, and empirical treatments prior to definitive diagnosis, which places an additional burden on both patients and healthcare providers [36]. In the context of our cohort, in which more than half on the patients experienced a diagnostic delay of 10 years or more, these findings underscore the clinical urgency of earlier recognition and diagnosis of endometriosis.

In the Polish setting, this delay may also result from the late implementation of a treatment-coordination system in highly specialized centers nationwide, and from the fact that reimbursement of endometriosis-dedicated procedures by the national payer only began in 2025. Nevertheless, other factors also appear to play a role, such as symptom suppression following the initiation of hormonal treatment and/or analgesic combination therapy, cultural factors, and the level of societal awareness of the disease, including among physicians.

Endometriosis is a hormone-dependent disease; therefore, it is reasonable to investigate the correlation between the onset of early symptoms and the beginning of hormonal activity in young girls.

Conducting an in-depth analysis of subjective parameters based on a questionnaire-based history does not guarantee comparability of the data reported by patients more than ten or even several decades after the onset of their first menstrual bleedings; nevertheless, for statistical purposes, such an analysis was undertaken in the patients included in the study.

Our results indicate that the age at menarche, in none of the analyzed age groups, correlates with the time at which the diagnosis was made, whether expressed as the overall diagnosis time or primary diagnosis time.

The lack of clear association between the age at menarche in our study and the development of endometriosis is consistent with other reports [37,38], although a metanalysis of 45 publications from 1980 to 2011 found an association between the risk of early menarche (<12 years) and the development of endometriosis [39], and a meta-analysis covering the years 2000–2020 confirmed this association specifically in countries with lower economic growth [40]. The presence of a correlation between early menarche and the development of endometriosis is consistent with the theory of retrograde menstruation, which applies to 10–15% of women of reproductive age, and points to the coincidence of genetic, epigenetic, environmental, and immunological factors in disease development [41]. The absence of an association between early menarche and disease severity in our cohort should be interpreted with caution, as no direct assessment of socioeconomic status was performed in the present study, and this finding may reflect factors other than national economic development.

Among the analyzed characteristics of the first menstrual bleedings—painfulness, heaviness, and regularity—only painfulness showed a statistically significant association. The duration of symptoms currently attributed to endometriosis was longest among patients whose first menstrual bleedings were associated with pain that was difficult to tolerate, and shortest among patients whose first menstrual bleedings were painless. An association between severe menstrual pain at the time of the first bleedings is one of the most reported features among patients who will later be diagnosed with endometriosis [42,43].

In the analysis of the relationship between the patient’s age at menarche and disease severity assessed intraoperatively using the rASRM scale, no correlation was found, which is consistent with other reports [38].

No correlation was found either between the patient’s age at diagnosis, or the duration of symptoms currently attributed to the onset of endometriosis, and disease severity as assessed by the rASRM scale. In accordance with numerous reports, the patient’s age at symptom onset, symptom severity, and disease duration do not correlate with disease severity as assessed by the rASRM scale [44,45,46].

However, regarding the relationship between the age at menarche and lesion localization, the study found that, according to the #ENZIAN classification, localization P (Peritoneum) and T (Tube) was highest among patients who reached menarche at 15–17 years of age, and lowest among patients who reached menarche at 7–11 years of age. A possible biological explanation for this finding may relate to the theory of retrograde menstruation [40], as both the peritoneal cavity and the fallopian tubes lie directly along the anatomical pathway of retrograde menstrual flow; differences in the pattern or volume of early menstrual cycles associated with a later age at menarche could theoretically influence the degree of retrograde seeding at these specific sited. This interpretation remains speculative and warrants further investigation. These results should nevertheless be interpreted with caution given the sample size and the absence of correction for multiple comparisons across the eleven tests performed in this analysis; accordingly, this finding should be regarded as exploratory rather than confirmatory (see false discovery rate correction discussed below). This association is not reflected in the literature.

The present study has several limitations. The retrospective design and the inclusion of a highly selected cohort of surgically treated patients with advanced, multi-organ endometriosis limit the generalizability and external validity of the findings. The retrospective nature of the history taken regarding the pubertal period, often obtained decades after the events in question and in the context of a very advanced disease with a course spanning many years, introduces the potential for recall bias, which could not be formally corrected for in the present analysis. The large number of subgroup analyses performed without formal correction for multiple comparisons increases the risk of a type I error; to quantify this risk, a post-hoc Benjamini–Hochberg false discovery rate correction was applied across all 50 comparisons reported in Table 2, Table 3, Table 4, Table 5, Table 6 and Table 7; none of the nominally significant findings remained significant after this correction (FDR-adjusted q > 0.48), confirming that they should be interpreted as exploratory and hypothesis-generating rather than confirmatory. Additionally, the absence of a priori power calculations limits the strength of the negative findings, as the study may have been underpowered to detect true associations, particularly in smaller subgroups. Although the surgical team was blinded to the questionnaire data, minimizing the risk of assessment bias related to menstrual history, inter-rater reliability for the #ENZIAN classification was not formally evaluated, which may have introduced some variability in disease staging. Accordingly, the results should be regarded as complementary to broader studies, and the conclusions drawn in this study are, for the most part, consistent with those reported in other publications.

## 5. Conclusions

The present study does not demonstrate a direct correlation between the patient’s age at menarche and the onset of the first symptoms that patients associate with the beginning of endometriosis, nor between the age at menarche and disease severity as assessed by the rASRM classification. At the same time, however, it does not exclude the existence of such associations. Owing to the highly complex and often decades-long course of the disease, its numerous and varied symptoms, and the individual, subjective perceptions of patients, establishing a definitive diagnosis can be considerably difficult. Before drawing definitive conclusions regarding the presence of the correlations discussed above, further research should consider the potential influence of modifying factors on the relationship between the primary cause of endometriosis (etiology) and the time to diagnosis. Several theories concerning the causes of endometriosis suggest the possible existence of different disease variants, upon which modifying factors are superimposed. Theories regarding the genetic or developmental basis of the disease may be substantially modified by environmental, cultural, or social factors. The additional influence of modifying factors—such as lifestyle (diet, physical activity), patients’ psychological makeup (personality traits, self-esteem, pain threshold), the organization of the healthcare system (accessibility, continuity, quality), social awareness (the level of knowledge among patients and medical professionals), and the impact of other health problems, comorbidities, or patients’ therapeutic decisions on the disease course (e.g., the use of medications affecting hormonal function for reasons unrelated to endometriosis)—may determine the actual time at which patients associate the onset of their first symptoms with endometriosis or the time to diagnosis by a physician.

## Figures and Tables

**Figure 1:**
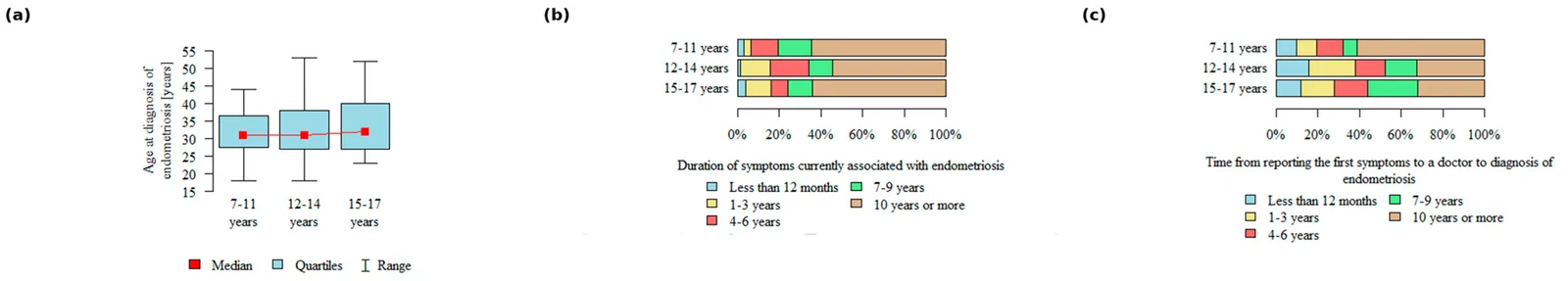
Age at diagnosis and diagnostic timelines according to the age at menarche: (**a**) Age at diagnosis of endometriosis (years) according to the age at menarche group; (**b**) Duration of symptoms currently attributed to endometriosis according to age at menarche group; (**c**) Time from reporting the first symptoms to a doctor to the diagnosis of endometriosis according to age at menarche group.

**Figure 2:**
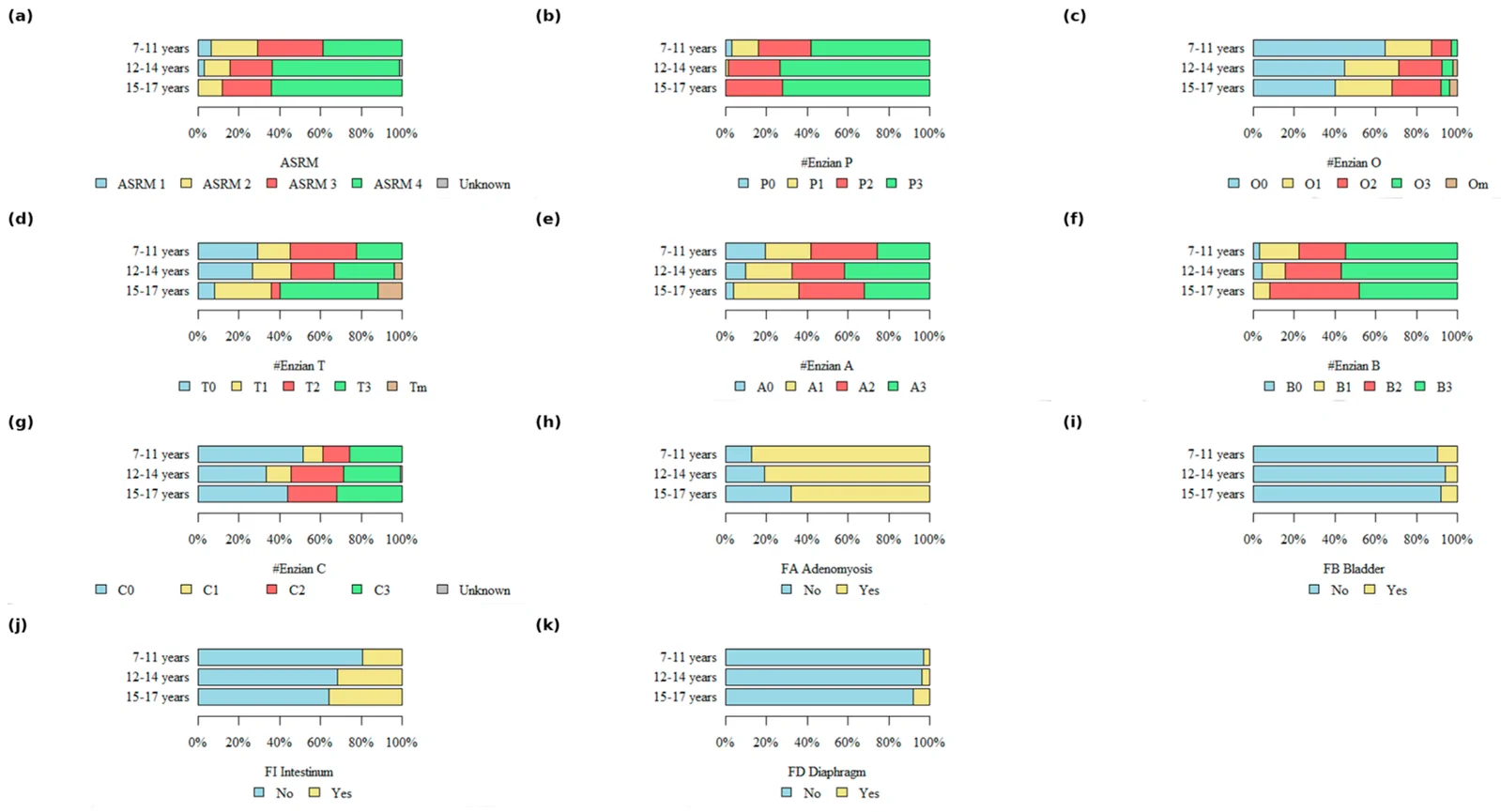
Disease severity according to the age at menarche: (**a**) rASRM stage according to age at menarche group; (**b**) #ENZIAN P score according to age at menarche group; (**c**) #ENZIAN O score according to age at menarche group; (**d**) #ENZIAN T score according to age at menarche group; (**e**) #ENZIAN A score according to age at menarche group; (**f**) #ENZIAN B score according to age at menarche group; (**g**) #ENZIAN C score according to age at menarche group; (**h**) Presence of adenomyosis (FA) according to age at menarche group; (**i**) Presence of bladder involvement (FB) according to age at menarche group; (**j**) Presence of intestinal involvement (FI) according to age at menarche group; (**k**) Presence of diaphragmatic involvement (FD) according to age at menarche group.

**Figure 3:**
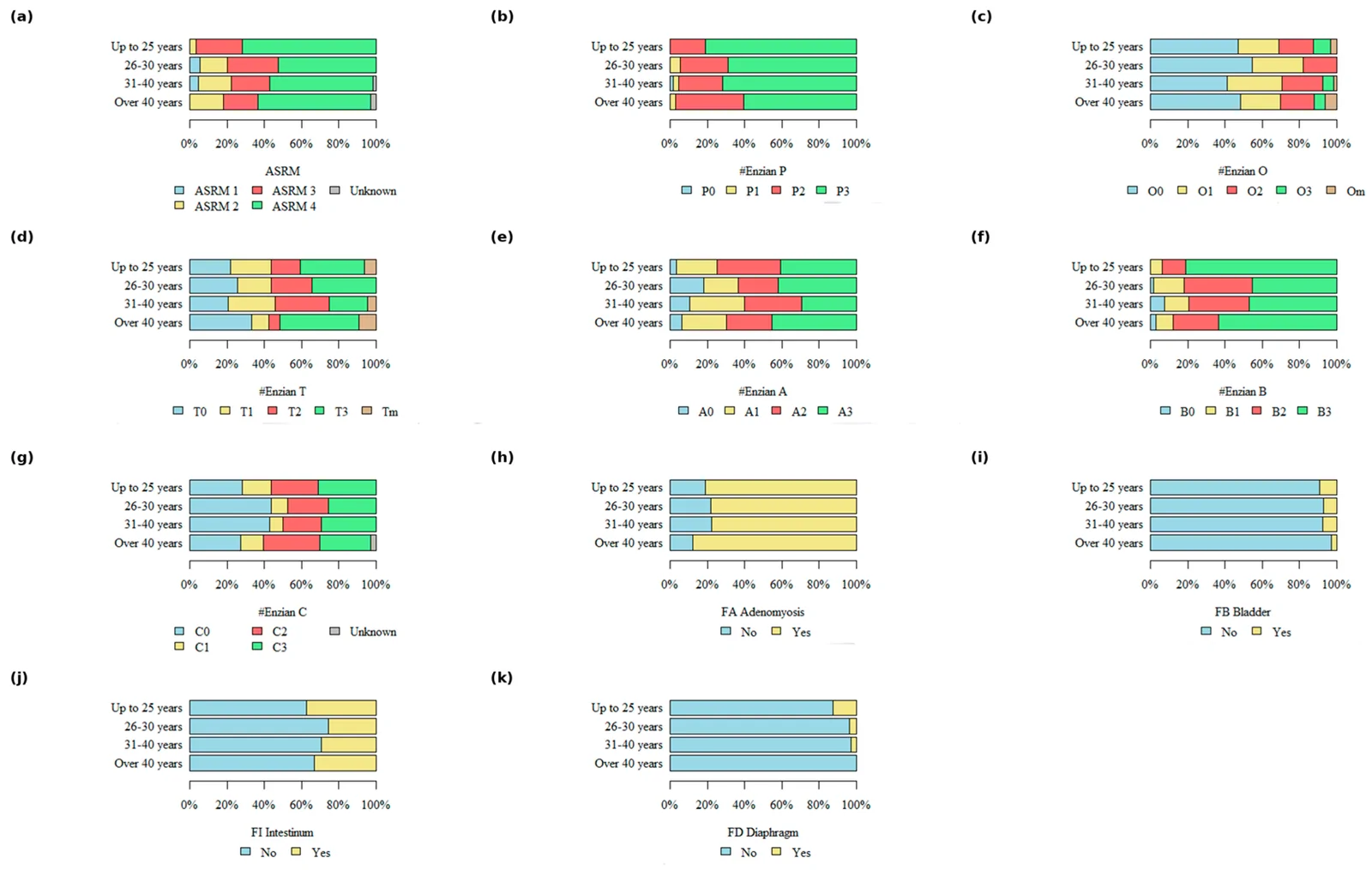
Disease severity according to age at diagnosis: (**a**) rASRM stage according to age diagnosis group; (**b**) #ENZIAN P score according to age at diagnosis group; (**c**) #ENZIAN O score according to age at diagnosis group; (**d**) #ENZIAN T score according to age at diagnosis group; (**e**) #ENZIAN A score according to age at diagnosis group; (**f**) #ENZIAN B score according to age at diagnosis group; (**g**) #ENZIAN C score according to age at diagnosis group; (**h**) Presence of adenomyosis (FA) according to age at diagnosis group; (**i**) Presence of bladder involvement (FB) according to age at diagnosis group; (**j**) Presence of intestinal involvement (FI) according to age at diagnosis group; (**k**) Presence of diaphragmatic involvement (FD) according to age at diagnosis group.

**Figure 4:**
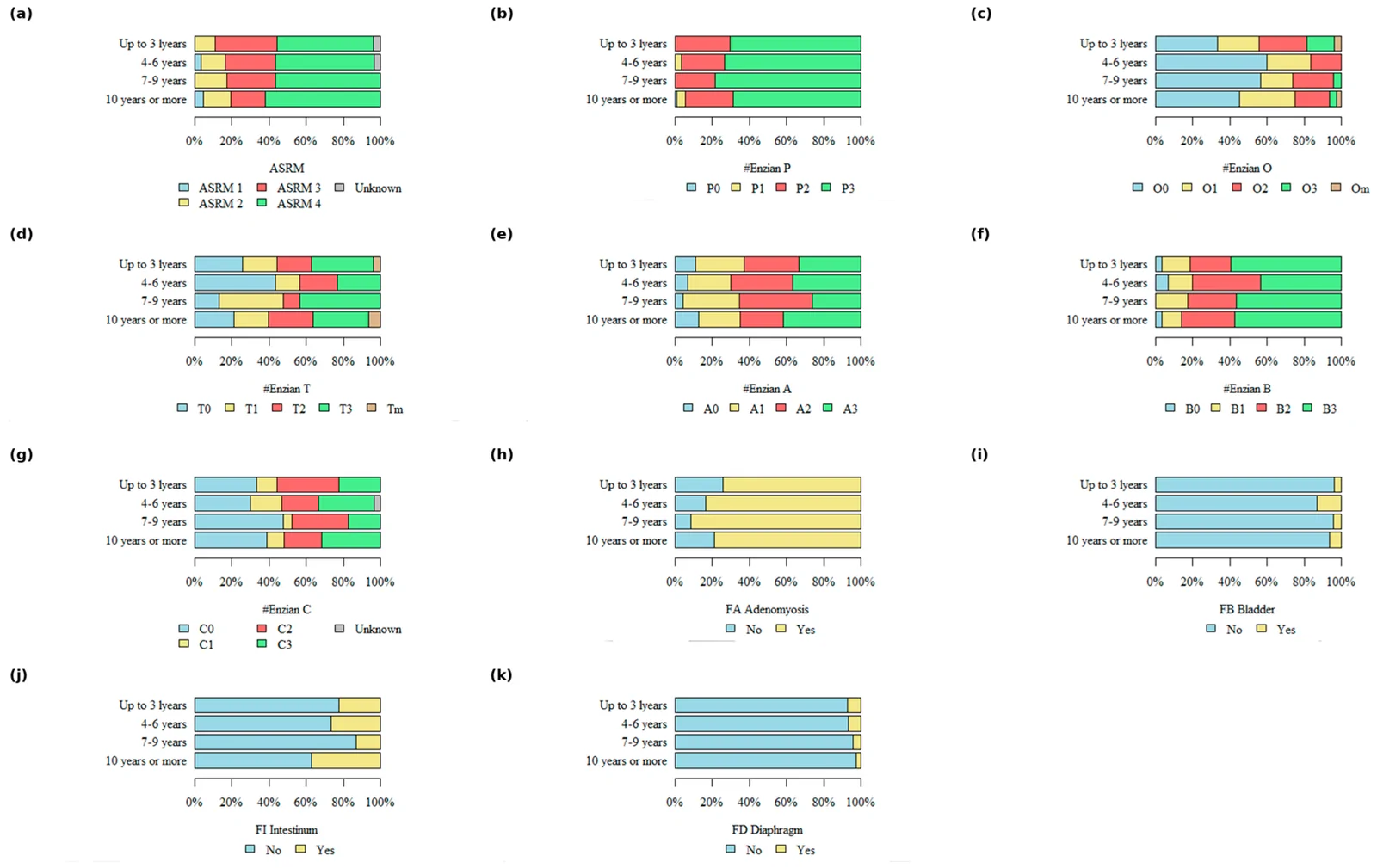
Disease severity according to the duration of symptoms currently attributed to endometriosis: (**a**) rASRM stage; (**b**) #ENZIAN P score; (**c**) #ENZIAN O score; (**d**) #ENZIAN T score; (**e**) #ENZIAN A score; (**f**) #ENZIAN B score; (**g**) #ENZIAN C score; (**h**) Presence of adenomyosis (FA); (**i**) Presence of bladder involvement (FB); (**j**) Presence of intestinal involvement (FI); (**k**) Presence of diaphragmatic involvement (FD)—each according to the duration of symptoms currently attributed to endometriosis.

**Figure 5:**
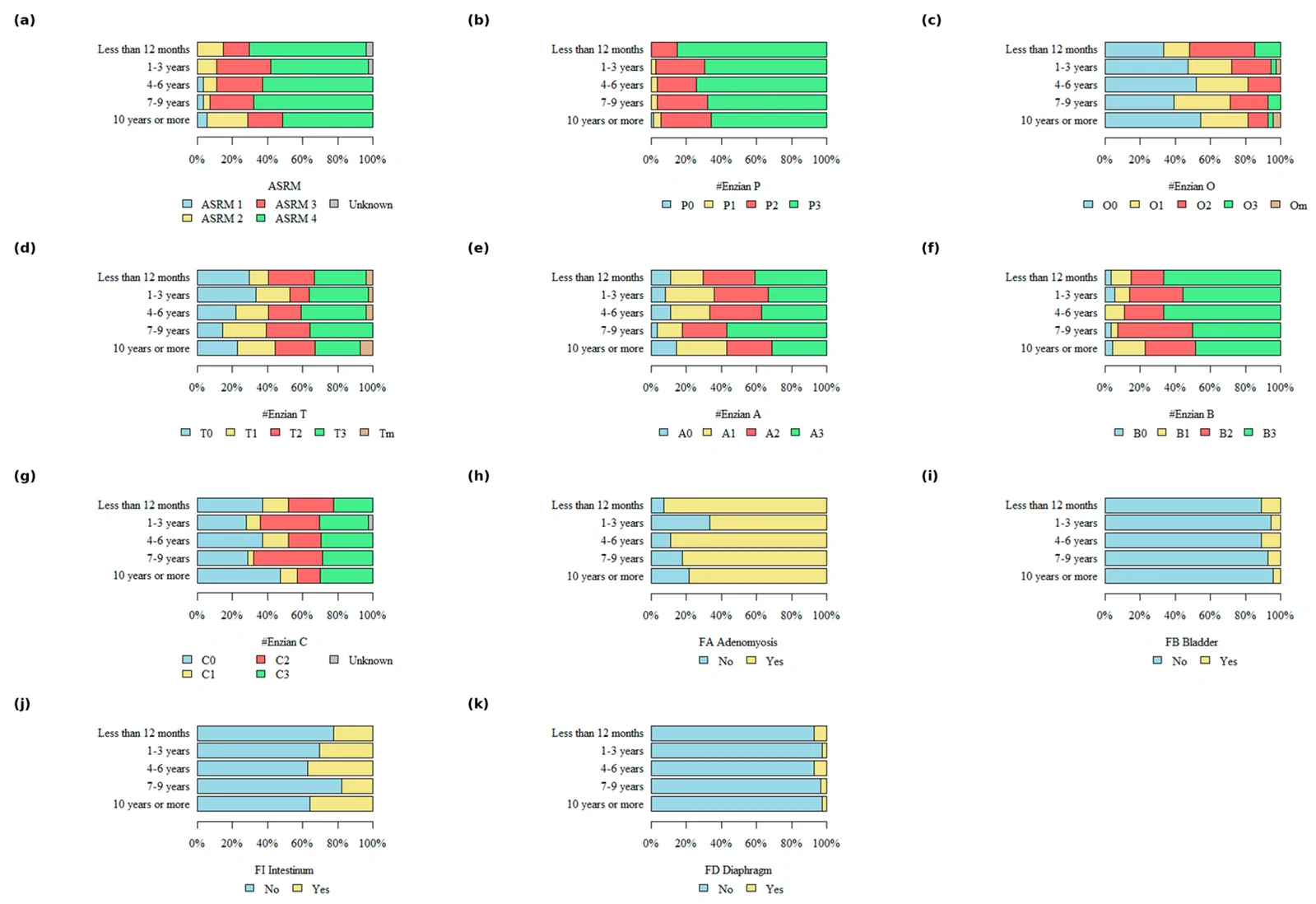
Disease severity according to the time from reporting the first symptoms to a physician to the diagnosis of endometriosis: (**a**) rASRM stage; (**b**) #ENZIAN P score; (**c**) #ENZIAN O score; (**d**) #ENZIAN T score; (**e**) #ENZIAN A score; (**f**) #ENZIAN B score; (**g**) #ENZIAN C score; (**h**) Presence of adenomyosis (FA); (**i**) Presence of bladder involvement (FB); (**j**) Presence of intestinal involvement (FI); (**k**) Presence of diaphragmatic involvement (FD)—each according to the time from reporting first symptoms to the diagnosis of endometriosis.

**Figure 6:**
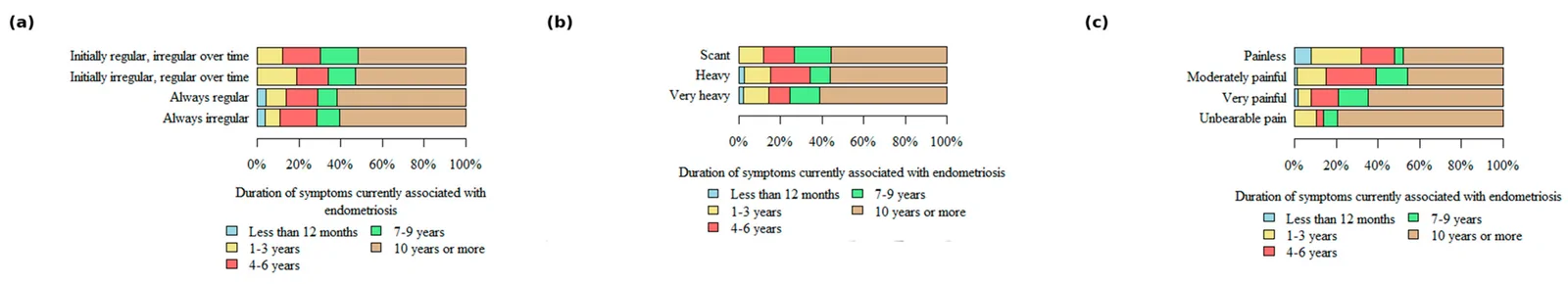
Duration of symptoms currently attributed to endometriosis according to the characteristics of the first menstrual bleedings: (**a**) Regularity of the first menstrual bleedings; (**b**) Heaviness of the first menstrual bleedings; (**c**) Painfulness of the first menstrual bleedings.

**Table 1:** Patients’ Characteristics.

Parameter		Total (N = 188)
Age [years]	Mean (SD)	37.84 (6.76)
	Median (quartiles)	38 (33–43)
	Range	22–54
	n	188
BMI	Thinness	3 (1.60%)
	Underweight	12 (6.38%)
	Normal weight	123 (65.43%)
	Overweight	37 (19.68%)
	Obesity	13 (6.91%)
	Obesity, class II	0 (0.00%)
	Obesity, class III	0 (0.00%)
Education	Primary	1 (0.53%)
	Vocational	8 (4.26%)
	Secondary	21 (11.17%)
	Higher	158 (84.04%)
Professional activity	Student	1 (0.53%)
	Employed	166 (88.30%)
	Unemployed	20 (10.64%)
	Pensioner	1 (0.53%)
Age at first menstruation [years]	Mean (SD)	12.78 (1.53)
	Median (quartiles)	13 (12–14)
	Range	8–17
	n	188
First menstruation	7–11 years	31 (16.49%)
	12–14 years	132 (70.21%)
	15–17 years	25 (13.30%)
Regularity of first menstruations	Initially regular, irregular over time	33 (17.55%)
	Initially irregular, regular over time	53 (28.19%)
	Always regular	73 (38.83%)
	Always irregular	28 (14.89%)
	Unknown	1 (0.53%)
Heaviness of first menstrual bleeding	Scant	34 (18.09%)
	Heavy	105 (55.85%)
	Very heavy	49 (26.06%)
Painfulness of first menstrual bleeding	Painless	25 (13.30%)
	Moderately painful	72 (38.30%)
	Very painful	62 (32.98%)
	Unbearable pain	29 (15.43%)
Age at diagnosis of endometriosis [years]	Mean (SD)	32.47 (7.27)
	Median (quartiles)	31 (27–38)
	Range	18–53
	n	188
Duration of symptoms currently associated with endometriosis	Less than 12 months	4 (2.13%)
	1–3 years	23 (12.23%)
	4–6 years	30 (15.96%)
	7–9 years	23 (12.23%)
	10 years or more	108 (57.45%)
Time from reporting the first symptoms to a doctor to diagnosis of endometriosis	Less than 12 months	27 (14.36%)
	1–3 years	36 (19.15%)
	4–6 years	27 (14.36%)
	7–9 years	28 (14.89%)
	10 years or more	70 (37.23%)
Duration of pain associated with endometriosis	No pain	6 (3.19%)
	1–4 days in a month	31 (16.49%)
	5–9 days in a month	35 (18.62%)
	10–14 days in a month	41 (21.81%)
	15 or more days in a month	75 (39.89%)
Pain in the lower pelvic area	No	4 (2.13%)
	Yes	184 (97.87%)
Intensity of pain in the most severe symptoms of pain in the lower pelvic region [NRS]	Mean (SD)	8.17 (2.13)
	Median (quartiles)	9 (7–10)
	Range	0–10
	n	188
Intensity of pain in the most severe symptoms of pain in the lower pelvic region	No pain (0)	4 (2.13%)
	Mild pain (1–4)	7 (3.72%)
	Medium pain (5–7)	37 (19.68%)
	Severe pain (8–10)	140 (74.47%)
Deep endometriosis	Non-DE	10 (5.32%)
	DE	178 (94.68%)

**Table 2:** Age at Menarche in Relation to the Timing of First Symptoms and Disease Duration.

Parameter		7–11 Years (N = 31)	12–14 Years (N = 132)	15–17 Years (N = 25)	*p*
Age at diagnosis of endometriosis [years]	Mean (SD)	31.74 (7.09)	32.36 (7.12)	33.96 (8.34)	*p* = 0.758
	Median (quartiles)	31 (27.5–36.5)	31 (27–38)	32 (27–40)	
	Range	18–44	18–53	23–52	
	n	31	132	25	
Duration of symptoms currently associated with endometriosis	Less than 12 months	1 (3.23%)	2 (1.52%)	1 (4.00%)	*p* = 0.503
	1–3 years	1 (3.23%)	19 (14.39%)	3 (12.00%)	
	4–6 years	4 (12.90%)	24 (18.18%)	2 (8.00%)	
	7–9 years	5 (16.13%)	15 (11.36%)	3 (12.00%)	
	10 years or more	20 (64.52%)	72 (54.55%)	16 (64.00%)	
Time from reporting the first symptoms to a doctor to diagnosis of endometriosis	Less than 12 months	3 (9.68%)	21 (15.91%)	3 (12.00%)	*p* = 0.167
	1–3 years	3 (9.68%)	29 (21.97%)	4 (16.00%)	
	4–6 years	4 (12.90%)	19 (14.39%)	4 (16.00%)	
	7–9 years	2 (6.45%)	20 (15.15%)	6 (24.00%)	
	10 years or more	19 (61.29%)	43 (32.58%)	8 (32.00%)	

*p*—Qualitative variables: chi-squared test or Fisher’s exact test, applied according to expected cell counts (Fisher’s exact test used when more than 20% of cells had an expected count below 5). Quantitative variables: Kruskal–Wallis test.

**Table 3:** Age at Menarche in Relation to Disease Severity Assessed by the rASRM and #ENZIAN Classifications.

Parameter		First Menstruation			*p*
		7–11 Years (N = 31)	12–14 Years (N = 132)	15–17 Years (N = 25)	
ASRM	ASRM 1	2 (6.45%)	4 (3.03%)	0 (0.00%)	*p* = 0.23
	ASRM 2	7 (22.58%)	17 (12.88%)	3 (12.00%)	
	ASRM 3	10 (32.26%)	27 (20.45%)	6 (24.00%)	
	ASRM 4	12 (38.71%)	82 (62.12%)	16 (64.00%)	
	Unknown	0 (0.00%)	2 (1.52%)	0 (0.00%)	
#ENZIAN P	P0	1 (3.23%)	0 (0.00%)	0 (0.00%)	*p* = 0.029 *
	P1	4 (12.90%)	2 (1.52%)	0 (0.00%)	
	P2	8 (25.81%)	33 (25.00%)	7 (28.00%)	
	P3	18 (58.06%)	97 (73.48%)	18 (72.00%)	
#ENZIAN O	O0	20 (64.52%)	59 (44.70%)	10 (40.00%)	*p* = 0.637
	O1	7 (22.58%)	35 (26.52%)	7 (28.00%)	
	O2	3 (9.68%)	28 (21.21%)	6 (24.00%)	
	O3	1 (3.23%)	7 (5.30%)	1 (4.00%)	
	Om	0 (0.00%)	3 (2.27%)	1 (4.00%)	
#ENZIAN T	T0	9 (29.03%)	35 (26.52%)	2 (8.00%)	*p* = 0.023 *
	T1	5 (16.13%)	25 (18.94%)	7 (28.00%)	
	T2	10 (32.26%)	28 (21.21%)	1 (4.00%)	
	T3	7 (22.58%)	39 (29.55%)	12 (48.00%)	
	Tm	0 (0.00%)	5 (3.79%)	3 (12.00%)	
#ENZIAN A	A0	6 (19.35%)	13 (9.85%)	1 (4.00%)	*p* = 0.375
	A1	7 (22.58%)	30 (22.73%)	8 (32.00%)	
	A2	10 (32.26%)	34 (25.76%)	8 (32.00%)	
	A3	8 (25.81%)	55 (41.67%)	8 (32.00%)	
#ENZIAN B	B0	1 (3.23%)	6 (4.55%)	0 (0.00%)	*p* = 0.527
	B1	6 (19.35%)	15 (11.36%)	2 (8.00%)	
	B2	7 (22.58%)	36 (27.27%)	11 (44.00%)	
	B3	17 (54.84%)	75 (56.82%)	12 (48.00%)	
#ENZIAN C	C0	16 (51.61%)	44 (33.33%)	11 (44.00%)	*p* = 0.259
	C1	3 (9.68%)	16 (12.12%)	0 (0.00%)	
	C2	4 (12.90%)	34 (25.76%)	6 (24.00%)	
	C3	8 (25.81%)	37 (28.03%)	8 (32.00%)	
	Unknown	0 (0.00%)	1 (0.76%)	0 (0.00%)	
FA Adenomyosis	No	4 (12.90%)	25 (18.94%)	8 (32.00%)	*p* = 0.185
	Yes	27 (87.10%)	107 (81.06%)	17 (68.00%)	
FB Bladder	No	28 (90.32%)	124 (93.94%)	23 (92.00%)	*p* = 0.595
	Yes	3 (9.68%)	8 (6.06%)	2 (8.00%)	
FI Intestinum	No	25 (80.65%)	90 (68.18%)	16 (64.00%)	*p* = 0.319
	Yes	6 (19.35%)	42 (31.82%)	9 (36.00%)	
FD Diaphragm	No	30 (96.77%)	127 (96.21%)	23 (92.00%)	*p* = 0.45
	Yes	1 (3.23%)	5 (3.79%)	2 (8.00%)	

*p*—Qualitative variables: chi-squared test or Fisher’s exact test, applied according to expected cell counts (Fisher’s exact test used when more than 20% of cells had an expected count below 5). Quantitative variables: Kruskal–Wallis test. “Unknown” denotes cases in which the classification could not be determined intraoperatively; these cases were included as a separate category in the statistical comparison. * Statistically significant (*p* < 0.05).

**Table 4:** Age at Diagnosis in Relation to Disease Severity Assessed by the rASRM and #ENZIAN Classifications.

Parameter		Age at Diagnosis of Endometriosis				*p*
		Up to 25 Years (N = 32)	26–30 Years (N = 55)	31–40 Years (N = 68)	Over 40 Years (N = 33)	
ASRM	ASRM 1	0 (0.00%)	3 (5.45%)	3 (4.41%)	0 (0.00%)	*p* = 0.412
	ASRM 2	1 (3.12%)	8 (14.55%)	12 (17.65%)	6 (18.18%)	
	ASRM 3	8 (25.00%)	15 (27.27%)	14 (20.59%)	6 (18.18%)	
	ASRM 4	23 (71.88%)	29 (52.73%)	38 (55.88%)	20 (60.61%)	
	Unknown	0 (0.00%)	0 (0.00%)	1 (1.47%)	1 (3.03%)	
#ENZIAN P	P0	0 (0.00%)	0 (0.00%)	1 (1.47%)	0 (0.00%)	*p* = 0.667
	P1	0 (0.00%)	3 (5.45%)	2 (2.94%)	1 (3.03%)	
	P2	6 (18.75%)	14 (25.45%)	16 (23.53%)	12 (36.36%)	
	P3	26 (81.25%)	38 (69.09%)	49 (72.06%)	20 (60.61%)	
#ENZIAN O	O0	15 (46.88%)	30 (54.55%)	28 (41.18%)	16 (48.48%)	*p* = 0.573
	O1	7 (21.88%)	15 (27.27%)	20 (29.41%)	7 (21.21%)	
	O2	6 (18.75%)	10 (18.18%)	15 (22.06%)	6 (18.18%)	
	O3	3 (9.38%)	0 (0.00%)	4 (5.88%)	2 (6.06%)	
	Om	1 (3.12%)	0 (0.00%)	1 (1.47%)	2 (6.06%)	
#ENZIAN T	T0	7 (21.88%)	14 (25.45%)	14 (20.59%)	11 (33.33%)	*p* = 0.077
	T1	7 (21.88%)	10 (18.18%)	17 (25.00%)	3 (9.09%)	
	T2	5 (15.62%)	12 (21.82%)	20 (29.41%)	2 (6.06%)	
	T3	11 (34.38%)	19 (34.55%)	14 (20.59%)	14 (42.42%)	
	Tm	2 (6.25%)	0 (0.00%)	3 (4.41%)	3 (9.09%)	
#ENZIAN A	A0	1 (3.12%)	10 (18.18%)	7 (10.29%)	2 (6.06%)	*p* = 0.301
	A1	7 (21.88%)	10 (18.18%)	20 (29.41%)	8 (24.24%)	
	A2	11 (34.38%)	12 (21.82%)	21 (30.88%)	8 (24.24%)	
	A3	13 (40.62%)	23 (41.82%)	20 (29.41%)	15 (45.45%)	
#ENZIAN B	B0	0 (0.00%)	1 (1.82%)	5 (7.35%)	1 (3.03%)	*p* = 0.05
	B1	2 (6.25%)	9 (16.36%)	9 (13.24%)	3 (9.09%)	
	B2	4 (12.50%)	20 (36.36%)	22 (32.35%)	8 (24.24%)	
	B3	26 (81.25%)	25 (45.45%)	32 (47.06%)	21 (63.64%)	
#ENZIAN C	C0	9 (28.12%)	24 (43.64%)	29 (42.65%)	9 (27.27%)	*p* = 0.776
	C1	5 (15.62%)	5 (9.09%)	5 (7.35%)	4 (12.12%)	
	C2	8 (25.00%)	12 (21.82%)	14 (20.59%)	10 (30.30%)	
	C3	10 (31.25%)	14 (25.45%)	20 (29.41%)	9 (27.27%)	
	Unknown	0 (0.00%)	0 (0.00%)	0 (0.00%)	1 (3.03%)	
FA Adenomyosis	No	6 (18.75%)	12 (21.82%)	15 (22.06%)	4 (12.12%)	*p* = 0.656
	Yes	26 (81.25%)	43 (78.18%)	53 (77.94%)	29 (87.88%)	
FB Bladder	No	29 (90.62%)	51 (92.73%)	63 (92.65%)	32 (96.97%)	*p* = 0.781
	Yes	3 (9.38%)	4 (7.27%)	5 (7.35%)	1 (3.03%)	
FI Intestinum	No	20 (62.50%)	41 (74.55%)	48 (70.59%)	22 (66.67%)	*p* = 0.667
	Yes	12 (37.50%)	14 (25.45%)	20 (29.41%)	11 (33.33%)	
FD Diaphragm	No	28 (87.50%)	53 (96.36%)	66 (97.06%)	33 (100.00%)	*p* = 0.096
	Yes	4 (12.50%)	2 (3.64%)	2 (2.94%)	0 (0.00%)	

*p*—Qualitative variables: chi-squared test or Fisher’s exact test, applied according to expected cell counts (Fisher’s exact test used when more than 20% of cells had an expected count below 5). Quantitative variables: Kruskal–Wallis test. “Unknown” denotes cases in which the classification could not be determined intraoperatively; these cases were included as a separate category in the statistical comparison.

**Table 5:** Duration of Symptoms Currently Attributed to Endometriosis in Relation to Disease Severity.

Parameter		Duration of Symptoms Currently Associated with Endometriosis				*p*
		Up to 3 Years (N = 27)	4–6 Years (N = 30)	7–9 Years (N = 23)	10 Years or More (N = 108)	
ASRM	ASRM 1	0 (0.00%)	1 (3.33%)	0 (0.00%)	5 (4.63%)	*p* = 0.765
	ASRM 2	3 (11.11%)	4 (13.33%)	4 (17.39%)	16 (14.81%)	
	ASRM 3	9 (33.33%)	8 (26.67%)	6 (26.09%)	20 (18.52%)	
	ASRM 4	14 (51.85%)	16 (53.33%)	13 (56.52%)	67 (62.04%)	
	Unknown	1 (3.70%)	1 (3.33%)	0 (0.00%)	0 (0.00%)	
#ENZIAN P	P0	0 (0.00%)	0 (0.00%)	0 (0.00%)	1 (0.93%)	*p* = 0.974
	P1	0 (0.00%)	1 (3.33%)	0 (0.00%)	5 (4.63%)	
	P2	8 (29.63%)	7 (23.33%)	5 (21.74%)	28 (25.93%)	
	P3	19 (70.37%)	22 (73.33%)	18 (78.26%)	74 (68.52%)	
#ENZIAN O	O0	9 (33.33%)	18 (60.00%)	13 (56.52%)	49 (45.37%)	*p* = 0.307
	O1	6 (22.22%)	7 (23.33%)	4 (17.39%)	32 (29.63%)	
	O2	7 (25.93%)	5 (16.67%)	5 (21.74%)	20 (18.52%)	
	O3	4 (14.81%)	0 (0.00%)	1 (4.35%)	4 (3.70%)	
	Om	1 (3.70%)	0 (0.00%)	0 (0.00%)	3 (2.78%)	
#ENZIAN T	T0	7 (25.93%)	13 (43.33%)	3 (13.04%)	23 (21.30%)	*p* = 0.147
	T1	5 (18.52%)	4 (13.33%)	8 (34.78%)	20 (18.52%)	
	T2	5 (18.52%)	6 (20.00%)	2 (8.70%)	26 (24.07%)	
	T3	9 (33.33%)	7 (23.33%)	10 (43.48%)	32 (29.63%)	
	Tm	1 (3.70%)	0 (0.00%)	0 (0.00%)	7 (6.48%)	
#ENZIAN A	A0	3 (11.11%)	2 (6.67%)	1 (4.35%)	14 (12.96%)	*p* = 0.726
	A1	7 (25.93%)	7 (23.33%)	7 (30.43%)	24 (22.22%)	
	A2	8 (29.63%)	10 (33.33%)	9 (39.13%)	25 (23.15%)	
	A3	9 (33.33%)	11 (36.67%)	6 (26.09%)	45 (41.67%)	
#ENZIAN B	B0	1 (3.70%)	2 (6.67%)	0 (0.00%)	4 (3.70%)	*p* = 0.863
	B1	4 (14.81%)	4 (13.33%)	4 (17.39%)	11 (10.19%)	
	B2	6 (22.22%)	11 (36.67%)	6 (26.09%)	31 (28.70%)	
	B3	16 (59.26%)	13 (43.33%)	13 (56.52%)	62 (57.41%)	
#ENZIAN C	C0	9 (33.33%)	9 (30.00%)	11 (47.83%)	42 (38.89%)	*p* = 0.601
	C1	3 (11.11%)	5 (16.67%)	1 (4.35%)	10 (9.26%)	
	C2	9 (33.33%)	6 (20.00%)	7 (30.43%)	22 (20.37%)	
	C3	6 (22.22%)	9 (30.00%)	4 (17.39%)	34 (31.48%)	
	Unknown	0 (0.00%)	1 (3.33%)	0 (0.00%)	0 (0.00%)	
FA Adenomyosis	No	7 (25.93%)	5 (16.67%)	2 (8.70%)	23 (21.30%)	*p* = 0.441
	Yes	20 (74.07%)	25 (83.33%)	21 (91.30%)	85 (78.70%)	
FB Bladder	No	26 (96.30%)	26 (86.67%)	22 (95.65%)	101 (93.52%)	*p* = 0.543
	Yes	1 (3.70%)	4 (13.33%)	1 (4.35%)	7 (6.48%)	
FI Intestinum	No	21 (77.78%)	22 (73.33%)	20 (86.96%)	68 (62.96%)	*p* = 0.086
	Yes	6 (22.22%)	8 (26.67%)	3 (13.04%)	40 (37.04%)	
FD Diaphragm	No	25 (92.59%)	28 (93.33%)	22 (95.65%)	105 (97.22%)	*p* = 0.419
	Yes	2 (7.41%)	2 (6.67%)	1 (4.35%)	3 (2.78%)	

*p*—Qualitative variables: chi-squared test or Fisher’s exact test, applied according to expected cell counts (Fisher’s exact test used when more than 20% of cells had an expected count below 5). Quantitative variables: Kruskal-Wallis test. “Unknown” denotes cases in which the classification could not be determined intraoperatively; these cases were included as a separate category in the statistical comparison.

**Table 6:** Time from First Presenting to a Physician with Initial Symptoms to the Diagnosis of Endometriosis in Relation to Disease Severity.

Parameter		Time from Reporting the First Symptoms to a Doctor to Diagnosis of Endometriosis					*p*
		Less than 12 Months (N = 27)	1–3 Years (N = 36)	4–6 Years (N = 27)	7–9 Years (N = 28)	10 Years or More (N = 70)	
ASRM	ASRM 1	0 (0.00%)	0 (0.00%)	1 (3.70%)	1 (3.57%)	4 (5.71%)	*p* = 0.305
	ASRM 2	4 (14.81%)	4 (11.11%)	2 (7.41%)	1 (3.57%)	16 (22.86%)	
	ASRM 3	4 (14.81%)	11 (30.56%)	7 (25.93%)	7 (25.00%)	14 (20.00%)	
	ASRM 4	18 (66.67%)	20 (55.56%)	17 (62.96%)	19 (67.86%)	36 (51.43%)	
	Unknown	1 (3.70%)	1 (2.78%)	0 (0.00%)	0 (0.00%)	0 (0.00%)	
#ENZIAN P	P0	0 (0.00%)	0 (0.00%)	0 (0.00%)	0 (0.00%)	1 (1.43%)	*p* = 0.941
	P1	0 (0.00%)	1 (2.78%)	1 (3.70%)	1 (3.57%)	3 (4.29%)	
	P2	4 (14.81%)	10 (27.78%)	6 (22.22%)	8 (28.57%)	20 (28.57%)	
	P3	23 (85.19%)	25 (69.44%)	20 (74.07%)	19 (67.86%)	46 (65.71%)	
#ENZIAN O	O0	9 (33.33%)	17 (47.22%)	14 (51.85%)	11 (39.29%)	38 (54.29%)	*p* = 0.13
	O1	4 (14.81%)	9 (25.00%)	8 (29.63%)	9 (32.14%)	19 (27.14%)	
	O2	10 (37.04%)	8 (22.22%)	5 (18.52%)	6 (21.43%)	8 (11.43%)	
	O3	4 (14.81%)	1 (2.78%)	0 (0.00%)	2 (7.14%)	2 (2.86%)	
	Om	0 (0.00%)	1 (2.78%)	0 (0.00%)	0 (0.00%)	3 (4.29%)	
#ENZIAN T	T0	8 (29.63%)	12 (33.33%)	6 (22.22%)	4 (14.29%)	16 (22.86%)	*p* = 0.825
	T1	3 (11.11%)	7 (19.44%)	5 (18.52%)	7 (25.00%)	15 (21.43%)	
	T2	7 (25.93%)	4 (11.11%)	5 (18.52%)	7 (25.00%)	16 (22.86%)	
	T3	8 (29.63%)	12 (33.33%)	10 (37.04%)	10 (35.71%)	18 (25.71%)	
	Tm	1 (3.70%)	1 (2.78%)	1 (3.70%)	0 (0.00%)	5 (7.14%)	
#ENZIAN A	A0	3 (11.11%)	3 (8.33%)	3 (11.11%)	1 (3.57%)	10 (14.29%)	*p* = 0.718
	A1	5 (18.52%)	10 (27.78%)	6 (22.22%)	4 (14.29%)	20 (28.57%)	
	A2	8 (29.63%)	11 (30.56%)	8 (29.63%)	7 (25.00%)	18 (25.71%)	
	A3	11 (40.74%)	12 (33.33%)	10 (37.04%)	16 (57.14%)	22 (31.43%)	
#ENZIAN B	B0	1 (3.70%)	2 (5.56%)	0 (0.00%)	1 (3.57%)	3 (4.29%)	*p* = 0.507
	B1	3 (11.11%)	3 (8.33%)	3 (11.11%)	1 (3.57%)	13 (18.57%)	
	B2	5 (18.52%)	11 (30.56%)	6 (22.22%)	12 (42.86%)	20 (28.57%)	
	B3	18 (66.67%)	20 (55.56%)	18 (66.67%)	14 (50.00%)	34 (48.57%)	
#ENZIAN C	C0	10 (37.04%)	10 (27.78%)	10 (37.04%)	8 (28.57%)	33 (47.14%)	*p* = 0.28
	C1	4 (14.81%)	3 (8.33%)	4 (14.81%)	1 (3.57%)	7 (10.00%)	
	C2	7 (25.93%)	12 (33.33%)	5 (18.52%)	11 (39.29%)	9 (12.86%)	
	C3	6 (22.22%)	10 (27.78%)	8 (29.63%)	8 (28.57%)	21 (30.00%)	
	Unknown	0 (0.00%)	1 (2.78%)	0 (0.00%)	0 (0.00%)	0 (0.00%)	
FA Adenomyosis	No	2 (7.41%)	12 (33.33%)	3 (11.11%)	5 (17.86%)	15 (21.43%)	*p* = 0.082
	Yes	25 (92.59%)	24 (66.67%)	24 (88.89%)	23 (82.14%)	55 (78.57%)	
FB Bladder	No	24 (88.89%)	34 (94.44%)	24 (88.89%)	26 (92.86%)	67 (95.71%)	*p* = 0.626
	Yes	3 (11.11%)	2 (5.56%)	3 (11.11%)	2 (7.14%)	3 (4.29%)	
FI Intestinum	No	21 (77.78%)	25 (69.44%)	17 (62.96%)	23 (82.14%)	45 (64.29%)	*p* = 0.35
	Yes	6 (22.22%)	11 (30.56%)	10 (37.04%)	5 (17.86%)	25 (35.71%)	
FD Diaphragm	No	25 (92.59%)	35 (97.22%)	25 (92.59%)	27 (96.43%)	68 (97.14%)	*p* = 0.657
	Yes	2 (7.41%)	1 (2.78%)	2 (7.41%)	1 (3.57%)	2 (2.86%)	

*p*—Qualitative variables: chi-squared test or Fisher’s exact test, applied according to expected cell counts (Fisher’s exact test used when more than 20% of cells had an expected count below 5). Quantitative variables: Kruskal-Wallis test. “Unknown” denotes cases in which the classification could not be determined intraoperatively; these cases were included as a separate category in the statistical comparison.

**Table 7:** Regularity, Heaviness, and Painfulness of the First Menstrual Bleedings in Relation to the Onset of the First Symptoms the Patient Associates with Endometriosis.

Parameter	Group	Duration of Symptoms Currently Associated with Endometriosis					*p*
		Less than 12 Months	1–3 Years	4–6 Years	7–9 Years	10 Years or More	
Regularity of first menstruations	Initially regular, irregular over time (N = 33)	0 (0.00%)	4 (12.12%)	6 (18.18%)	6 (18.18%)	17 (51.52%)	*p* = 0.729
	Initially irregular, regular over time (N = 53)	0 (0.00%)	10 (18.87%)	8 (15.09%)	7 (13.21%)	28 (52.83%)	
	Always regular (N = 73)	3 (4.11%)	7 (9.59%)	11 (15.07%)	7 (9.59%)	45 (61.64%)	
	Always irregular (N = 28)	1 (3.57%)	2 (7.14%)	5 (17.86%)	3 (10.71%)	17 (60.71%)	
Heaviness of first menstrual bleeding	Scant (N = 34)	0 (0.00%)	4 (11.76%)	5 (14.71%)	6 (17.65%)	19 (55.88%)	*p* = 0.847
	Heavy (N = 105)	3 (2.86%)	13 (12.38%)	20 (19.05%)	10 (9.52%)	59 (56.19%)	
	Very heavy (N = 49)	1 (2.04%)	6 (12.24%)	5 (10.20%)	7 (14.29%)	30 (61.22%)	
Painfulness of first menstrual bleeding	Painless (N = 25)	2 (8.00%)	6 (24.00%)	4 (16.00%)	1 (4.00%)	12 (48.00%)	*p* = 0.024 *
	Moderately painful (N = 72)	1 (1.39%)	10 (13.89%)	17 (23.61%)	11 (15.28%)	33 (45.83%)	
	Very painful (N = 62)	1 (1.61%)	4 (6.45%)	8 (12.90%)	9 (14.52%)	40 (64.52%)	
	Unbearable pain (N = 29)	0 (0.00%)	3 (10.34%)	1 (3.45%)	2 (6.90%)	23 (79.31%)	

*p*—Qualitative variables: chi-squared test or Fisher’s exact test, applied according to expected cell counts (Fisher’s exact test used when more than 20% of cells had an expected count below 5). Quantitative variables: Kruskal–Wallis test. * Statistically significant (*p* < 0.05).

## Data Availability

No new data were created or analyzed in this study. Data sharing is not applicable to this article.

## Abbreviations

The following abbreviations are used in this manuscript:

ASRM	American Society for Reproductive Medicine
rASRM	Revised American Society for Reproductive Medicine classification
#ENZIAN	ENZIAN classification system for deep infiltrating endometriosis
BMI	Body Mass Index
SD	Standard Deviation
SEF	Stiftung Endometriose-Forschung
